# Adolescents and Transition-Age Youths with Intellectual Disabilities in Saudi Arabia: An Exploration of Parental Perspectives

**DOI:** 10.3390/bs16010066

**Published:** 2026-01-01

**Authors:** Mohaned G. Abed, Todd K. Shackelford

**Affiliations:** 1 Department of Special Education, Faculty of Education, King Abdulaziz University, Jeddah 21589, Saudi Arabia; mabed@kau.edu.sa; 2 Department of Psychology, Oakland University, Rochester, MI 48306, USA

**Keywords:** intellectual disability, social experiences, social support, adolescents, parents, challenges, needs, Saudi Arabia, social inclusion, family quality of life

## Abstract

The current study explores the social experiences of adolescent and transition-age youths with intellectual disabilities (IDs) and the support mechanisms available to these groups in Saudi Arabia. This study adopts a qualitative methodology with a semi-structured interview constituting the data collection method involving 13 parents with children aged between 11 and 19 years, a critical adolescent period and transition to early adulthood. The results suggest that family, caregivers, community, friendships, and healthcare providers play important roles that impact the quality of life for these groups. The main challenges identified include health-related issues, employment challenges, educational barriers, insufficient services, inadequate community participation, and limited social relationships, with special emphasis on obstacles linked to transition during the 18 to 19-year period when youths must navigate transfers from pediatric to adult services and changes associated with legal rights. This study highlights several reasons it is important to increase awareness and education, while also continuing to improve support systems aimed at dealing with both transition challenges and adolescent needs. The results further illustrate that although support from family provides the foundation for care, systemic changes are needed to promote social inclusion and reduce stigma during critical development periods. The current study contributes to the limited research related to IDs in the context of the Middle East, with special reference to Saudi Arabia. Finally, the discussion highlights several insights that are culturally specific for the development of policy and provision of services associated with the transition from adolescence to early adulthood.

## 1. Introduction

As lifelong conditions, intellectual disabilities (IDs) are characterized by considerable limitations in intellectual functioning and adaptive behaviors. This definition is supported by the American Psychiatric Association (2013), which adds that the onset of this condition occurs before the age of 18 years. Maulik et al. (2011) and Olusanya et al. (2020) report that 1–2% of the global population is affected by IDs. El-Hazmi et al. (2003) report that the prevalence of IDs among children in Saudi Arabia is 8.9%. According to the same author, most cases fall into the moderate to severe category.

It is not just the impacts of IDs that affect these individuals’ wellbeing. Other important elements include social and environmental factors, which can limit access to fulfilling activities and valued social roles (Werner & Scior, 2016). Limited opportunities for education and employment, stigmatization, and social exclusion are some of the leading difficulties contributing to entrenched disadvantage (Grogan et al., 2019; Emerson & Hatton, 2014; Sawant et al., 2025). Other factors that make these disadvantages more detrimental include limited support networks and community participation, which can result in loneliness and poor physical and mental health outcomes (Alexandra et al., 2018; Emerson & Hatton, 2014).

Social inclusion is a primary determinant of quality of life for people with ID. This factor includes access to opportunities for personal development and growth, the development of social relationships, and meaningful participation in social life (Hall, 2009; Cummins & Lau, 2003). It has been consistently demonstrated that social inclusion nurtures confidence, self-esteem, and happiness, which are all factors that have a positive effect on mental health as they have the potential to reduce isolation and loneliness (Werner & Scior, 2016; Sawant et al., 2025). However, the achievement of social inclusion is difficult, especially for adolescents with IDs. This conclusion is supported by studies that have found that around 44% of adolescents with IDs report loneliness, which is higher than rates observed in the general population (Alexandra et al., 2018; Al-Shathri et al., 2024).

Parents whose children have IDs often have a lifelong caregiving role. Usually, these parents face difficulties, including financial burdens and navigating complex service systems (Apanasionok et al., 2025; Lafferty et al., 2016). Because of these responsibilities, such parents often have higher levels of depression, anxiety, and stress, especially when there are insufficient support services (Aunos et al., 2008). Parental experiences are also shaped by cultural factors, particularly in societies with inadequate disability services and strong traditional roles, such as in Saudi Arabia, which is the focus of the current research (Alnahdi, 2024; Hadidi & Al Khateeb, 2015).

## 2. The Context of Saudi Arabia

Regarding disability rights, Saudi Arabia has made significant legislative progress. Alnahdi (2024) reports that the country recently ratified the Convention on the Rights of Persons with Disabilities (CRPD) and enacted the Rights of Persons with Disabilities Act. Abobaker (2024) analyzed the kingdom’s systematic approach to the development of disability-rights legislation. The same scholar addressed requisite reforms in the educational system intended to ensure inclusive practices and equal access for individuals with IDs. Saudi Arabia’s Vision 2030 emphasizes the wellbeing and community participation of individuals with disabilities, which reflects a national commitment to the promotion of equality and inclusion (Alnahdi, 2024). Notwithstanding this progress, people with IDs still face societal barriers, including social exclusion, insufficient access to services, and negative societal attitudes (Hadidi & Al Khateeb, 2015; Yousef, 2018).

In Saudi Arabia, research addressing IDs is limited. Hadidi and Al Khateeb (2015) and Alnahdi (2024) note that most researchers focus on educational experiences as opposed to comprehensive social experiences. A recent bibliometric analysis indicates that IDs, mental health, and quality of life are among the leading recent themes in disability research in Saudi Arabia, which can be interpreted as an increase in interest in this field among Saudi Arabian researchers (Albarrati et al., 2025). In their bibliometric analysis of disability in Saudi Arabia, Albarrati et al. (2025) identified the main research trends, gaps in research, and future research directions that can inform the development of evidence-based policies and interventions for people with IDs. Notwithstanding, there remains a considerable gap in understanding the lived experiences of adolescents with IDs and their families. Alnahdi (2024) concludes that this challenge is particularly linked to social and cultural factors.

In Saudi society, the traditional family structure emphasizes familial responsibility for people with disabilities. Hadidi and Al Khateeb (2015) and Alnahdi (2024) note that this often results in lifelong caregiving arrangements in which parents, especially fathers, assume primary decision-making roles. Such a cultural context leads to a distinct dynamic, which influences the way families navigate the difficulties and opportunities associated with IDs (Alnahdi, 2024).

## 3. Research Objectives

The aim of this study was to address the research gaps noted above. We achieved this by exploring the lived experiences of adolescents with IDs in Saudi Arabia by collecting data from parents, including fathers, whose roles are often pivotal in the process by which families in the country’s cultural context make decisions (Hadidi & Al Khateeb, 2015). The study employed qualitative methodology. Regarding the specific data collection method, the study used semi-structured interviews, selected because they permit in-depth exploration of social experiences and cultural nuances that cannot easily be captured with self-report survey techniques. Through the examination of challenges and support mechanisms from the perspective of parents, the study sought to inform practice and policy, facilitating greater social inclusion and wellbeing for Saudi children with IDs. The study’s specific objectives were to:

Explore the primary challenges that individuals with IDs face in Saudi Arabia and how these are perceived by their parents.

Examine the roles played by different support systems, including caregivers, the community, friendships, family, and healthcare providers.

Gather recommendations from parents for improving social support and provision of services for adolescents with IDs in the Saudi context.

The results of this culturally specific study may inform similar cultural contexts across the Arab world, especially given similarities in family structures and disability-related difficulties in the region (Hadidi & Al Khateeb, 2015).

## 4. Literature Review

**Prevalence and Global Context.** The American Psychiatric Association (2013) defines intellectual disability (ID) as a condition characterized by considerable limitations in intellectual functioning and adaptive behavior, beginning before the individual reaches 18 years of age. Regarding global prevalence, estimates range from below 1% in the developed world to greater than 10% in low-and middle-income countries due to factors like environmental risks, limited access to healthcare, and poverty (Maulik et al., 2011; Olusanya et al., 2020). The influence of complex interactions among social, environmental, and biological factors on ID prevalence is also emphasized by the World Health Organization (WHO, 2011).

A systematic review conducted by McKenzie et al. (2016) focusing on ID prevalence and incidence revealed methodological challenges in epidemiological research. The same scholars also emphasized the importance of standardized diagnostic criteria and wide-ranging data collection systems for accurate estimation of prevalence. It has been concluded that prevalence rates in the Middle East are higher than global averages. Alnahdi (2024) adds that contributing factors include the environment, limited prenatal care in many regions, and consanguineous marriages. The estimated disability prevalence rate of about 9% in Saudi Arabia reflects these regional patterns, underscoring the significance of developing a wide-ranging support system for people with disabilities and their families (Alnahdi, 2024).

**Social Inclusion and Quality of Life**. Social inclusion is a multi-layered concept that includes acceptance, participating in fulfilling social activities, and developing and maintaining meaningful social connections (Cummins & Lau, 2003; Hall, 2009). For people with IDs, social inclusion is an important determinant of quality of life, affecting self-esteem, emotional wellbeing, and satisfaction with life (Werner & Scior, 2016; Sawant et al., 2025). It has been consistently demonstrated that social inclusion has a large impact on quality-of-life outcomes for people with IDs, in comparison to other domains such as material and physical wellbeing (Morisse et al., 2013).

When social inclusion is promoted, individuals with IDs are provided with an opportunity to make meaningful contributions to their communities (Staunton et al., 2023; Sawant et al., 2025). Notwithstanding, achieving social inclusion requires addressing numerous barriers, including societal attitudes, insufficient support services, limited accessibility, and social stigma (Werner & Scior, 2016; Yazbeck et al., 2004). A systematic review conducted by Bigby (2012) focused on social inclusion for people with IDs and challenging behaviors. The study concluded that although inclusion initiatives show promise to be effective, they require wide-ranging support systems and community acceptance. This study underscores the challenges of achieving genuine social inclusion, especially for people whose support needs are complex.

Saudi Arabian researchers have recently investigated educational quality of life for students with IDs. For instance, Hassan and Al-Rasheed (2024) examined the perspectives of special education teachers on quality-of-life factors for students with IDs. The study revealed that there are several important concerns relating to long-term outcomes, educational accommodations, and social inclusion. Their findings highlight the critical role played by institutional support and educational accommodations in the promotion of desirable educational experiences for students with IDs in Saudi Arabia.

**Relationships and Social Networks.** The wellbeing of people with IDs can be enhanced by strong social networks. Harrison et al. (2021) and Forrester-Jones et al. (2006) note that these networks can provide opportunities for social engagement, practical assistance, and emotional support to people with IDs. Previous studies have documented that adolescents with IDs often have limited social networks compared to their non-disabled peers (Harrison et al., 2021; Alexandra et al., 2018). Limited social networks can lead to social isolation and decreased opportunities for community integration (Kamstra et al., 2015).

For people with IDs, the quality of social relationships is particularly important. Harrison et al. (2021) and Forrester-Jones et al. (2006) report that positive relationships contribute to higher self-esteem. Alexandra et al. (2018) and Sawant et al. (2025) concluded that negative social experiences, such as social exclusion, discrimination, and rejection, can result in loss of self-confidence, psychological distress, and withdrawal from social activities. An examination of the social networks of people with mild learning disabilities by Van Asselt-Goverts et al. (2015) concluded that quality of life and relationship satisfaction were impacted by how supportive and diverse social connections are.

**Support Needs and Family Experiences**. Families of people with IDs face distinct difficulties that often change over time (Lafferty et al., 2016). It is common for parents to accept caregiving responsibilities that extend beyond the roles of parents whose children do not have these challenges. Examples of these responsibilities are presented by Apanasionok et al. (2025) and include long-term planning, care coordination, and advocacy. Apanasionok et al. (2025) and Alnahdi (2024) add that these roles can affect parental wellbeing, financial resources, and family functioning.

An analysis of studies focusing on family quality of life reveals differences in the way different family members experience the difficulties associated with IDs (Alnahdi, 2024). In Arab cultures, the family member who often experiences unique challenges related to a child’s IDs is the father. This is due to their traditional roles as decision-makers and providers in families, particularly in cultures that link masculine identity with financial success and professional achievement (Alnahdi, 2024; Hadidi & Al Khateeb, 2015).

Family experiences are impacted by cultural factors. Collectivist cultures, such as in Saudi Arabia, emphasize interdependence and family responsibility (Alnahdi, 2024; Hadidi & Al Khateeb, 2015). Even though this cultural emphasis on family support can facilitate robust informal support networks, it also has the potential to put more pressure on families and can limit behaviors related to seeking help when professional services are required (Alnahdi, 2024; Hadidi & Al Khateeb, 2015). A systematic review and meta-analysis of psychological interventions for parents of children with IDs conducted by Ranta et al. (2025) concluded that targeted support programs were effective in reducing parental stress and improving the manner in which families function.

**Vocational and Employment Challenges**. For many adolescents with IDs, employment is an important element influencing quality of life and social inclusion (Sawant et al., 2025; Staunton et al., 2023). Apart from financial benefits, gainful employment also makes available opportunities for community contribution, skill development, and social interaction (Staunton et al., 2023). However, there are often unique challenges faced by individuals with IDs regarding employment. Al-Shammari (2022) and Ojagbemi et al. (2022) identify several such challenges, including limited workplace accommodations, a dearth of suitable job training programs, employers’ attitudes and styles of management, discrimination, and social stigma. Often, these challenges are accompanied by insufficient transition services from school to work and inadequate supported employment programs (Al-Shammari, 2022).

Regarding the context of Saudi Arabia, even though the law requires that individuals with disabilities have equal opportunities for employment in the private and public sectors, there remain challenges related to implementation (Al-Shammari, 2022). An example of an attempt to address these challenges is the Sa3ee rehabilitation and employment initiative (Al-Shammari, 2022). Notwithstanding such efforts, evaluation research has identified remaining barriers such as limited employer awareness and a lack of training for students with IDs in secondary school (Al-Shammari, 2022). The perspectives of individuals with IDs regarding the Sa3ee program are explored by Almalki et al. (2022), who reported mixed experiences that bring to the fore the role of person-centered approaches in employment support services.

**Healthcare and Service Access**. For individuals with IDs and their families, access to appropriate healthcare and support services represents a key challenge (Apanasionok et al., 2025). Scott and Havercamp (2014) note that, compared to the general population, individuals with IDs face poorer healthcare outcomes, including premature mortality, chronic diseases, and higher rates of mental health conditions. Exacerbating these disparities are barriers to healthcare access, including inadequate service coordination, communication challenges, and lack of knowledge (Scott & Havercamp, 2014; Apanasionok et al., 2025).

For individuals with IDs and their families in Saudi Arabia, mental health considerations are particularly relevant. The King Salman Center for Disability Research (2024) reported greater mental health challenges among families caring for people with disabilities, including stress-related conditions, depression, and anxiety. Within this context, the role of caregivers is crucial as they often manage healthcare access and coordination (Apanasionok et al., 2025). Researchers also note that if healthcare workers are to be effective in fulfilling their roles, they need support in the form of emotional support, respite services, and training (Flynn et al., 2025). Both the outcomes for people with IDs and the wellbeing of caregivers are influenced by the existence and quality of caregiver support services (Apanasionok et al., 2025).

**Digital and Technological Inclusion**. The potential role of technology in boosting inclusion and social participation for individuals with IDs is highlighted in emerging research (Shpigelman, 2018; Caton & Chapman, 2016). Digital technologies, especially platforms for social media, make available opportunities for improving autonomy, establishing new relationships, and maintaining connections (Shpigelman, 2018). However, significant barriers remain for people with IDs in relation to digital inclusion, including concerns about safety, inadequate digital literacy support, and restricted access to technology (Chadwick et al., 2022).

During COVID-19, the limitations of technology-mediated social connections for individuals with IDs came to the fore (Embregts et al., 2021; McCausland et al., 2021). Although digital platforms made available important opportunities for maintaining social connections during lockdown, many people with IDs faced barriers to technology access and use, possibly worsening their social isolation (Chadwick et al., 2022). Studies have shown that for digital inclusion to be successful, there is a need for supportive staff with positive attitudes towards the use of technology, reliable access to the internet, and personalized equipment (Björquist & Tryggvason, 2023).

**Cultural Considerations in the Saudi Context**. Regarding understanding the experiences of people with IDs and their families, the cultural context of Saudi Arabia presents distinct challenges (Alnahdi, 2024; Hadidi & Al Khateeb, 2015). For example, religious beliefs, family structures, and traditional gender roles impact perceptions of disability and how it is addressed in Saudi Arabian society (Alnahdi, 2024; Hadidi & Al Khateeb, 2015). It is essential to understand these cultural factors if support services and interventions are to be effective. In pursuit of this understanding, recent studies in Saudi Arabia have explored parental experiences and family quality of life, providing insights into the distinct needs and challenges of families in Saudi Arabia (Alnahdi, 2024).

## 5. Methodology

### 5.1. Research Design

The aims of the current study were accomplished with qualitative research. The specific data collection method used was semi-structured interviews, which afforded the opportunity to capture the lived experiences of parents of adolescents and transition-age youth with IDs in Saudi Arabia (Lafferty et al., 2016). This qualitative approach allowed an in-depth exploration of the topic, affording a nuanced assessment of challenges and support systems, which aligns with recommendations for research in culturally specific contexts (Apanasionok et al., 2025). As a methodology, a qualitative paradigm makes it possible to secure rich, detailed data that informs theory and practice in a manner that would be more difficult with a large sample, self-report survey quantitative methodology (Hall, 2009).

### 5.2. Sampling

The study sample consisted of 13 Saudi parents of adolescents and transition-age youth with IDs. Specifically, seven fathers and six mothers comprised the study sample. All participants lived in Jeddah and were Saudi nationals. Eight participants hold bachelor’s degrees, and the other five hold secondary school certificates. Four participants were unemployed, whereas nine were employed. The participants’ ages ranged from 40 to 57 years. The age range of their children with IDs was 11 to 19 years. This age range was selected with the aim of capturing experiences during an important transition phase from adolescence to early adulthood, a time that Lafferty et al. (2016) and Hall (2009) report is characterized by the emergence of unique social and vocational challenges. Convenience sampling was employed for recruiting participants. The inclusion criteria required participants to have adolescents or transition-age youth with IDs, within the age range of 11 to 19 years; to be between the ages of 40 and 60 years; and to reside in Saudi Arabia. A summary of the available participant characteristics is presented in Table 1.

The decision to include both genders of parents as participants reflects an interest in capturing varied parental perspectives while also recognizing the distinct roles of Saudi family dynamics and the process of making decisions (Alnahdi, 2024; Hadidi & Al Khateeb, 2015). Fathers in Saudi Arabian society often have primary responsibility in external family affairs, including service coordination, educational planning, and healthcare decisions, whereas mothers usually have the responsibility of providing daily caregiving (Hadidi & Al Khateeb, 2015). From this cultural positioning, fathers and mothers can provide distinct insights into the challenges and resources available to families of individuals with IDs (Hadidi & Al Khateeb, 2015).

### 5.3. Procedures for Data Collection

Data for the present study were collected through semi-structured interviews conducted in Arabic. Each interview lasted approximately 35 min. In developing the interview guide, the researchers were informed by existing literature on family experiences around adolescents with IDs in Saudi Arabia (e.g., Alnahdi, 2024; Hadidi & Al Khateeb, 2015). The guide included questions addressing the challenges faced by adolescents and transition-age youth with IDs from parental perspectives, the roles played by healthcare providers, caregivers, the community, family, and friendships in supporting social experiences, and solicited recommendations on how social support systems can be improved. In designing the interview questions, the aim of the researchers was to elicit narratives about the experiences of participants while maintaining adequate structure to ensure consistency across interviews. With open-ended prompts, participants were encouraged to elaborate on their responses and provide specific examples of challenges and support experiences. Through the conversational character of the interviews, follow-up questions and clarifications were possible. After securing verbal, informed consent from participants, all interviews were audio-recorded and conducted at locations preferred by the participant to ensure confidentiality and participant comfort. The discussions were facilitated by a trained and experienced interviewer who is proficient in Arabic and accustomed to the Saudi cultural context. This ensured that the discussions were sensitive to cultural norms and expectations.

The researchers pilot-tested the interview guide with two parents before beginning the main data collection phase. This assisted in the assessment of elements such as cultural sensitivity and question appropriateness and clarity (Castillo-Montoya, 2016). With the pilot assessment, the researchers were able to refine both the questions and the interview techniques while also building confidence with the interview protocol (Malmqvist et al., 2019). Using feedback from the pilot assessment, the researchers made minor adjustments to the wording of the questions and sequencing to improve clarity and cultural appropriateness.

### 5.4. Ethical Considerations

Before beginning the data collection phase, the researchers received ethical approval from the King Abdulaziz University Research Ethics Committee (IPP:1008-324-2025). Each participant provided verbal informed consent after they were provided with information relating to the purpose of the study, procedures, and their rights as research participants. Each participant informed us that their participation was voluntary, and they had the right to withdraw from the study at any time without penalty. Throughout the study, confidentiality was maintained. The researcher stored audio recordings and transcripts securely, identifying information removed from the data files. Each participant was identified using a numerical identifier (Father 1, Mother 2, etc.). This was done with the aim of protecting privacy while still making it possible to track individual responses.

### 5.5. Data Analysis

The recordings from the interviews were transcribed verbatim in Arabic before they were translated into English. A qualified bilingual translator was hired for this task. The process of translating included back-translation verification, which aimed to ensure cultural appropriateness and accuracy (in terms of intended meaning) of the English version. The main themes associated with social experiences and support needs of adolescents and transition-age youth with IDs were identified using thematic analysis. NVivo 15 software for data management and analysis was employed (Braun & Clarke, 2006). The analysis followed established procedures for qualitative data analysis, as follows:

1. Repeated reading of transcripts to familiarize with the data;
2. Initial coding of meaningful units of text;
3. Identification of potential themes;
4. Reviewing and refining themes;
5. Defining and naming final themes;
6. Interpreting and reporting findings.

With the aim of ensuring intercoder reliability, a subset of the transcripts was independently coded by a second researcher using the same framework. Through discussion and consensus-building, discrepancies between coders were resolved. This was achieved through negotiating shared understanding of code definitions and applications (Cofie et al., 2022). This process consisted of scrutinizing the precise occasions of disagreements, discussing the reasoning behind the different code assessments, and arriving at a consensus through dialogue while retaining an audit trail of all discussions (O’Connor & Joffe, 2020). In the rare instances in which the two researchers failed to reach consensus, a third researcher experienced in the qualitative methodology was invited to provide an additional perspective and facilitate a resolution. The data analysis process was iterative, ensuring that the themes emanated from the data as opposed to being imposed by predetermined categories. Using this approach made it possible to identify culturally specific issues and experiences that may not have been captured using analytical frameworks that are more structured.

### 5.6. Methodological Rigor

Several strategies were adopted to improve the credibility and trustworthiness of the results. An important measure with regard to this was “member checking,” a process in which participants are requested to review the transcribed responses from their interview for accuracy. Peer debriefing sessions with the researchers, maintaining an audit trail documenting analytical decisions, and reflexive journaling to acknowledge researcher cultural positioning and biases were the other measures adopted (Lincoln & Guba, 1985). The research team’s cultural positioning was important given the study’s cross-cultural character. The lead researcher’s familiarity with the Arabic language and Saudi culture facilitated the building of rapport with participants and afforded comprehension of cultural nuances in the data (Hadidi & Al Khateeb, 2015).

## 6. Results

From the thematic analysis, three main domains characterizing the experiences of adolescents with IDs in Saudi Arabia were identified. The first relates to challenges faced by individuals and families. The second concerns sources of support and their impacts, and the third includes recommendations for improvements. In each domain, there are interconnected themes reflecting the realities of living with IDs in the Saudi context. The present study was conducted before the initiatives under Saudi Arabia’s Vision 2030 and the 2023 Saudi Law on the Rights of Persons with Disabilities (SLRPD) were implemented.

**Domain 1: Challenges**

**Social Isolation and Stigma**. A leading challenge emanating from the data collected for the study is social isolation. All participants described instances in which their children experienced discrimination and exclusion. These experiences are in keeping with acknowledged patterns of disability stigma in Saudi society, whereby traditional beliefs, coupled with a dearth of public awareness, result in marginalization. In relation to this, Father 1 reported how he watched his son struggle with isolation during a community event. He notes, “Despite his enthusiasm for participating, others ignored him due to his different communication style, leading to misunderstandings.” The father commented that this treatment did not just hurt his son’s feelings but also resulted in his son withdrawing from social activities. He continued, “My son often talked about feeling ignored, which caused him anxiety. This experience increased his feelings of marginalization and loneliness.” The same father reported that this led them to seek psychological support to assist his son in dealing with these negative feelings and improving his social participation.

In the same vein as Father 1, Father 3 also elaborated on the psychological effects of social exclusion. He said, “My son has faced bullying and social discrimination, and recently he was ridiculed by a group of his peers who mocked him in front of others.” According to the same father, this situation led to his son lacking confidence because he felt inadequate in relation to his social abilities, restricting his ability to effectively communicate with others. The same father reported that “This has led to difficulty trusting others, making lasting friendships, and feeling isolated.” He also adds that such difficulties make it challenging for his son to express his desires and needs.

In relation to isolation and stigma, Mother 2 spoke about how her son loves the company of others, “but the kids at school don’t play with him.” The mother stated that she can see how this “breaks her son’s spirit,” leading him to come home asking, “Why don’t they like me?” The mother said she does not know how to respond to this question. Mother 4 narrated a similar story: “Even at family gatherings, some relatives avoid talking to him, and the social rejection he feels affects his self-image.” She said that this has led to her son becoming more withdrawn and unwilling to speak in public. An analysis of the reports of these mothers and fathers shows wider patterns acknowledged in research relating to the social exclusion of individuals with IDs across the world (Alexandra et al., 2018; Sawant et al., 2025). They are also in keeping with findings specific to stigma experiences in Saudi Arabia.

**Professional and Educational Challenges**. Participants identified employment challenges faced by their children with IDs. This is consistent with recent research from Saudi Arabia documenting barriers in disability employment programs. For example, a study by Al-Mughirah (2024) concluded that, despite the Sa3ee program objectives, employees still report substantial difficulties regarding support for individuals with IDs. Examples of these challenges include insufficient accommodations by workplaces, limited employer engagement, and inadequate training. All participants commented that the dearth of suitable employment and educational opportunities reflects substantial challenges to the development and prospects of their children. Several participants noted that continuing educational and employment challenges were especially disheartening given recent legislative efforts in Saudi Arabia, including the Law on the Rights of Persons with Disabilities enacted in 2023. The law seeks to ensure that educational and employment opportunities are available to people with disabilities.

Regarding educational barriers, Father 5 reported that, “The education system was a major obstacle.” He reported an instance in which his son was excited about joining a vocational training program, only to discover that the program was not equipped to meet his son’s special needs. From this experience, the father concludes, “Isolating children with intellectual disabilities from other students hinders their integration.” He further adds that a dearth of suitable educational and training programs has resulted in his son being delayed academically and missing out on skills development opportunities.

Father 7 also emphasized the importance of employment. He shared the view that, “Work is not just a source of income; it is an essential part of human life. For my son, work gives him an opportunity to prove himself. This comes through learning new skills and a sense of accomplishment and pride. He needs to feel like he is part of society and able to contribute to its productivity.” In the same vein, Mother 6 reported that she attempted to enroll her son in a school but was told, “they weren’t prepared to accommodate his condition.” She reported that she had to resort to a private center that she could hardly afford. Talking about her son, Mother 8 revealed a challenge: “He wants to work, but no one is hiring him.” She says that even when employers are considering employing her son, as soon as they hear the word “intellectual disability,” they are reluctant.

It is clear from the experiences of these parents that even though Saudi Arabia’s Vision 2030 demands disability inclusion, people with disabilities face challenges. This is consistent with the results of previous studies that have identified employment barriers for individuals with disabilities (Al-Mughirah, 2024). These barriers include employer attitudes, stigma, and discrimination. This is even though Saudi Arabian statutes mandate that 4% of positions be reserved for qualified persons with disabilities (Al-Mughirah, 2024).

**Health and Healthcare Access Challenges**. For families of individuals with IDs, healthcare access emerged as a substantial challenge. Participant parents in the current study described both direct healthcare effects on their children and indirect effects on the parents’ wellbeing. An analysis of these challenges shows that they reflect wider healthcare accessibility issues acknowledged in previous disability research. In describing healthcare-related stress, Father 9 said, “The stress I face due to my son has had a significant impact on my own health.” He described a time when he had to take leave from work due to stress-related issues. He talked about how he went through an especially challenging period when his son’s condition worsened, which led to additional psychological and financial burdens. He adds, “The growing anxiety about his future and access to adequate healthcare remains a challenge.” The same father supports this view by providing an example in which specialized treatment was not available locally, which can result in delayed treatment and increased stress and anxiety about what might happen to his son. Speaking about the same issues, Mother 10 said, “My son’s condition worsened one night, and we couldn’t find a specialized clinic that was open. I felt so helpless. We had to drive two hours to another city.” Mother 12 tells a similar story, noting that “These recurring health crises are exhausting not only physically but also psychologically. I feel like I’m constantly on alert.” She reports that she has not slept through the night in years. From the accounts of parents relating to health and healthcare challenges, it can be noted that there are systemic issues related to the provision of healthcare for people with IDs. Some of the leading difficulties include geographical challenges in service availability, long waiting times, and insufficient specialist services. Even though the healthcare system in Saudi Arabia includes specialist services, the country is still faced with access challenges for people with disabilities—including IDs—especially in rural areas.

**Parental and Family Challenges**. Another theme that emerged is the impact of caregiving responsibilities on parents. In this regard, participants described healthcare-related, emotional, and financial challenges. These challenges are especially difficult for fathers, who usually carry the burden of serving as the traditional breadwinners. In this regard, and talking about his son, Father 13 reports that he faces the challenge of balancing care and work responsibilities, and he reports financial pressures due to the reduced working hours concomitant with caring for his son. Father 3 also emphasized time management and financial pressures.

An analysis of the interview responses from the mothers reveals challenges associated with emotional burdens and effects on their mental health in their struggles to support their children with IDs. For example, they spoke about chronic anxiety and feelings of sadness linked to dealing with the challenges of their children’s ID, particularly in a society where formal psychological support opportunities are not readily available or are difficult to access. In relation to this, Mother 2 commented, “I suffer from constant anxiety about my son’s future, especially when I think about his condition after my death. These thoughts haunt me day and night, affecting my sleep and mental health. We need ongoing psychological support services and family counselling.” Mother 6 spoke about the same reality, noting that she quit her job to care for her child on a full-time basis, leaving the family dependent on her husband’s income. She concludes, “It’s hard to balance everything—financially and emotionally”.

**Domain 2: Support Systems**

From the responses of the interview participants, family support emerged as a central pillar of care and wellbeing for individuals with IDs. These findings are consistent with Islamic cultural values, emphasizing that the family is responsible for caring for family members with disabilities. In this regard, mothers highlighted the role of family ties, particularly relationships between siblings, in supporting the child and strengthening their sense of belonging in the family. Talking about this, Mother 4 said, “His older sister always defends him, stands by him, helps him, and explains his condition to people.” The same mother also reports that the older sister is always learning about her brother’s condition, which results in her dealing with him with kindness and understanding. The mother concludes, “Sometimes I feel she is more aware and compassionate than some adolescents.”

Father 1 spoke about the role of family support, noting that it is essential as the family gives their son “unconditional love and closely monitors his health and education.” He says that family support constitutes the cornerstone of his family, which is accomplished through organizing regular family gatherings with the aim of ensuring that his son feels included. The same father reports that, “Even his cousins participate and make a special effort to include him in their activities, which clearly improves his mood.” He referenced the joy he saw on his son’s face when the family organized a birthday party for him.

Father 5 spoke about the challenges related to developing friendships. He described both the challenges and the positive impact when supportive friendships develop. In his own words, “As for friendships, whenever my son tried to interact with those his age, he would find himself in an awkward situation or face unintentional neglect.” The same father notes that this has an impact on self-confidence, which leads to his son feeling incapable of establishing genuine relationships, even though he has “pure intentions and a kind heart.” He concludes, “Recently, however, one of his close friends was extremely supportive, providing him with companionship that helped alleviate his feelings of isolation.” A similar experience was shared by Father 7, who spoke about the role played by extended family support. On the other hand, Mothers 4 and 6 highlighted the way siblings provided emotional support to their children with IDs.

**Caregiver and Healthcare Provider Support**. Even though participants agreed that the role of healthcare providers and caregivers is important in supporting individuals with IDs, they also noted that the quality and approach were inconsistent. From this finding, it can be noted that caregiver training and competency are crucial elements that Saudi Arabia’s specialized training programs for disability service providers seek to address. The participating parents spoke about how they play a central role in coordinating between medical and educational institutions, and they are frequently involved in efforts to find suitable therapeutic and educational opportunities, often depending on informal support groups, including those hosted by other parents of children with similar challenges.

Regarding the role mothers play, Mother 4 said, “I personally search for appropriate treatment centers, and I participate in WhatsApp groups with mothers of children with the same condition as my son to exchange advice. We sometimes take on the responsibility of raising awareness in the community and at school.” In the same vein, Mother 10 talked about her relief when her family finally found a therapist who understood how to work with her son. She says, “This changed everything. Before that, we tried three centers, and none of them worked.” Father 1 reports on the variability of caregiver support, stating, “One of the biggest challenges we face as families of children with mental disabilities is the lack of awareness among many caregivers in different institutions.” The same father lamented that most of the caregivers do not understand his son’s needs, which leads them to fail to deal with him in a way that suits his abilities and social and psychological needs. Similar accounts were provided by Father 9 and Mother 8, who referred to inconsistent caregiver quality. On the other hand, Mother 10 spoke about her positive experience with well-trained healthcare providers who understood the needs of adolescents with IDs.

**Community Support**. Participants identified community support as an important part of building self-confidence and nurturing a sense of belonging. This finding is specifically notable with reference to Saudi Arabia’s Vision 2030, which emphasizes reducing social stigma and building inclusive communities. Father 11 described a positive community experience: “Community support is essential to my son’s self-confidence and sense of belonging, so providing awareness programs in local communities is crucial, and highlights a positive community experience.” The same father said that community support has made a positive difference in his son’s life. He reports that a local organization hosted an inclusive festival that permitted attendees with IDs to display their talents, at which his son participated in a musical performance. Following that performance, Father 11 reports that his son received positive feedback from the community, and this boosted his sense of belonging and self-confidence.

In relation to community support, Mother 6 said, “We participated in an inclusive sports day event, and my son won a medal. The smile stayed on his face for days. It was the first time he felt proud of himself.” Notwithstanding these positive experiences, Mother 8 indicated, “There are still places that exclude our children, but I have also noticed a change. Some communities have started inviting us to join.” Mothers 6 and 10, and Father 9, also talked about other community events that successfully included their children, showing the positive impact of communities actively embracing inclusion.

**Domain 3: Recommendations for Improvement**

Based on the wide-ranging experiences shared by the 13 participants, the results of the present study suggest the following recommendations:

(1) In alignment with Saudi Arabia’s Vision 2030 education initiatives, establish a comprehensive and flexible education system personalized to individuals with IDs.
(2) Use targeted community awareness campaigns to reduce stigma and showcase Islamic principles of respect and inclusion.
(3) Improve public infrastructure and accessibility, leveraging accessibility mandates.
(4) Introduce specialized employment programs and vocational training in alignment with Saudi Arabia’s 4% employment mandate for persons with disabilities.
(5) Expand support services, including mentorship and counseling programs.
(6) Encourage greater collaboration among private sector organizations, the public sector, and NGOs.
(7) Improve training programs for caregivers to ensure high-quality, consistent support by service providers.

## 7. Discussion

The present study explored the lived experiences of adolescents with intellectual disabilities (IDs) and their parents in Saudi Arabia, from the perspectives of the parents. The aim of the study was to gain insight into the challenges, support systems, and recommendations for improving inclusion. An analysis of the findings unearthed complex realities that validate both global research conclusions and culturally specific policy applications.

### 7.1. Key Findings

From the thematic analysis, three domains characterizing the experiences of adolescents with IDs in Saudi Arabia were identified: Challenges faced by individuals and families, sources of support and their impacts, and recommendations for improvement. These domains reflect wide-ranging experiences that mirror both global trends and circumstances specific to Saudi Arabia. These findings emerge at an important point in Saudi Arabia’s disability rights advancements, including enaction of the 2023 Saudi Law on the Rights of Persons with Disabilities (SLRPD) and the inclusion initiatives of Vision 2030 (Authority of People with Disability, 2024). This evolving landscape of disability rights and accessibility in Saudi Arabia is documented by Ahmed (2024). The same author highlights the dynamic character of policy development and implementation challenges that continue to affect the lived experiences of people with disabilities and their families.

### 7.2. Social Isolation and Stigma in the Saudi Context

The social isolation experienced by the children of participants reflects established patterns of disability stigma. Every participant in the current study described instances of discrimination and social exclusion, corroborating the conclusions of researchers such as Hadidi and Al Khateeb (2015) and Jansen-van Vuuren and Aldersey (2020). The psychological impacts described by parents, including social withdrawal, depression, and anxiety, align with findings from scholars such as Alexandra et al. (2018), who found that almost half of people with intellectual and developmental disabilities experience loneliness. Within the Saudi context, these experiences are especially disheartening because the country is influenced by Islamic principles that emphasize respect, inclusion, and compassion for all individuals (Al-Aoufi et al., 2012).

### 7.3. Educational and Employment Barriers

Despite recent legislative advances, participating parents reported persistent vocational and educational challenges for their adolescents with IDs. Saudi Arabia’s Vision 2030 Human Capability Development Program consists of policies designed to facilitate access for students with disabilities to education and inclusive learning programs (Authority of People with Disability, 2024). Despite introduction of these and related recent programs, all participants referred to educational environments that are segregated and vocational opportunities that are limited. This suggests challenges with implementation, corroborating previous reports of the broader educational and social challenges in Arab countries (Hadidi & Al Khateeb, 2015). If one considers Saudi Arabia’s National Transformation Program initiatives, which include the Mowaamah Program that has certified over 2000 organizations to make available suitable work environments for individuals with disabilities, the employment barriers described by the participants are particularly notable (Authority of People with Disability, 2024).

### 7.4. Challenges with Healthcare and Support Services

The participants described healthcare accessibility issues, which are reflective of the wider difficulties in the provision of services for people with disabilities. For example, Father 3 described stress-related health impacts and delays in specialist treatments, examples that bring to the fore the gaps that the National Committee for Medical Rehabilitation aims to address (Authority of People with Disability, 2024). These findings corroborate conclusions from international research, which demonstrated that the implications of social support are far-reaching for the mental health of adolescents with IDs (Scott & Havercamp, 2014). The stress that the participating parents report is a reflection of often complex family dynamics in which the effects of disability extend beyond the individual to affect the entire family, requiring wide-ranging support approaches (Taylor et al., 2019).

### 7.5. Connections to Global Research and Cross-Cultural Perspectives

The findings of the present study illustrate consistency with international research on experiences with IDs while also identifying Saudi-specific manifestations. The employment challenges, educational barriers, and social isolation are in keeping with global patterns documented across diverse cultural contexts (Bloom, 2020). Research on cross-cultural disability in the Middle East–North Africa region suggests that local understandings of disability, care, and community responsibility make available viable alternatives to disability discourse dominated by the West (Bloom, 2020).

### 7.6. Study Limitations and Future Directions

The present study has several limitations. First, the sample is small, including just 13 individuals reporting on their lived experiences. Second, participants were members of families residing in a single city, Jeddah, a large metropolitan area that differs in many ways from much of the rural landscape in Saudi Arabia. Third, and by chance, the adolescents of the parents who participated in this research were all males. That is, there were no female adolescents represented in this study. It would be worth investigating whether parents of adolescent daughters with IDs report similar or different lived experiences. Fourth, the sample included more male than female parent participants. These sample characteristics may limit generalizability of the current results to other regions in Saudi Arabia and to other familial perspectives. Fifth, we note that we did not secure information on the siblings of the focal adolescents with IDs. Future work could profitably investigate parental perceptions (and sibling perceptions) of sibling relationships of adolescents with IDs. Finally, we acknowledge that much of what we present in this article focuses on the challenges facing adolescents with IDs. An equally important direction for future research is to identify how adolescents with IDs and their parents can successfully cope with or navigate these challenges. Based on these limitations, future researchers could prioritize diverse geographical representation, balanced gender perspectives, and longitudinal studies with the aim of tracking progress as Vision 2030 initiatives mature.

### 7.7. Recommendations for Saudi Arabia

Based on the findings of the study and the prevailing policy context within the framework inspired by Vision 2030, some recommendations can be made. These include more consistent implementation of existing legislation, cultural and religious integration, professional development, infrastructure development, employment initiative expansion, and research and data collection to inform policy and initiatives, as supported by previous scholars (Al-Aoufi et al., 2012; Authority of People with Disability, 2024).

## 8. Conclusions

The present study provided important insights into the lived experiences of individuals with IDs and their parents in Saudi Arabia at a time of transformation marked by the comprehensive framework inspired by the country’s Vision 2030, which seeks to ensure that people with disabilities are included socially, economically, and politically. From the findings, persistent challenges and developing opportunities are identified as Saudi Arabia implements ambitious initiatives addressing community integration, accessibility, healthcare, employment, and education. By successfully addressing attitudinal, cultural, and structural barriers that the present study identified, Saudi Arabia has the potential to realize its commitments to creating a society in which people with IDs are valued, supported, and empowered to make a meaningful contribution to the societies in which they live.

## Figures and Tables

**Table 1:** Summary of participant characteristics.

Participant	Gender	Education Level	Employment Status
1	Father	Bachelor	Employed
2	Mother	Bachelor	Not employed
3	Father	Bachelor	Employed
4	Mother	Secondary	Not employed
5	Father	Bachelor	Employed
6	Mother	Secondary	Not employed
7	Father	Bachelor	Employed
8	Mother	Secondary	Not employed
9	Father	Bachelor	Employed
10	Mother	Bachelor	Employed
11	Father	Bachelor	Employed
12	Mother	Secondary	Employed
13	Father	Secondary	Employed

Note: “Secondary” refers to secondary teaching certificate, requiring two years to complete.

## Data Availability

In accordance with our agreement with the study participants, and as approved by our Institutional Review Board (IRB)/Ethics Committee, the raw interview transcripts were not retained for sharing or inclusion as part of the research. Therefore, they are not provided. The informed consent stated that all responses would remain confidential, and that no transcripts or identifiable raw data would be stored, shared, or included in any publications. To discuss, please contact the first author.
